# Safe and Successful Yttrium-90 Resin Microsphere Radioembolization in a Heavily Pretreated Patient with Chemorefractory Colorectal Liver Metastases after Biliary Stent Placement above the Papilla

**DOI:** 10.1155/2014/921406

**Published:** 2014-12-17

**Authors:** Vlasios S. Sotirchos, Elena N. Petre, Karen T. Brown, Lynn A. Brody, Michael I. D'Angelica, Ronald P. DeMatteo, Nancy E. Kemeny, Constantinos T. Sofocleous

**Affiliations:** ^1^Department of Radiology, Interventional Radiology Service, Memorial Sloan Kettering Cancer Center, 1275 York Avenue, New York, NY 10065, USA; ^2^Department of Surgery, Hepatopancreatobiliary Service, Memorial Sloan Kettering Cancer Center, 1275 York Avenue, New York, NY 10065, USA; ^3^Department of Medicine, Gastrointestinal Oncology Service, Memorial Sloan Kettering Cancer Center, 1275 York Avenue, New York, NY 10065, USA

## Abstract

We report a case of safe and successful yttrium-90 resin microsphere radioembolization in a patient with a long history of multiple recurrent colon cancer hepatic metastases progressing after hepatic resections, hepatic arterial chemotherapy, and multiple regimens of systemic chemotherapy. One month prior to radioembolization, a biliary stent was placed above the level of the ampulla to relieve tumor-related biliary obstruction and normalize bilirubin levels.

## 1. Introduction

Yttrium-90 (^90^Y) radioembolization or selective internal radiation therapy (SIRT) with resin microspheres is an FDA approved therapy for patients with colorectal cancer liver metastases (CLM) who are not candidates for hepatic resection or ablation but have adequate liver function and a satisfactory performance status [1]. Often, patients present for SIRT in the salvage setting with progression of disease after several prior treatments, including surgery, ablation, and/or chemotherapy with cytotoxic and biologic agents [2–5]. We report a case of chemorefractory CLM progressing after hepatic resections, hepatic arterial pump chemotherapy (HAC), and multiple regimens of systemic chemotherapy that was successfully treated with SIRT after biliary stenting above the papilla to relieve obstructive hyperbilirubinemia.

## 2. Case Presentation

A 75-year-old man presenting ten years earlier with synchronous metastatic colorectal cancer (T3N0M1) and multiple comorbidities was referred to our department for image-guided therapy due to progressive metastatic liver disease.

After initial diagnosis, the patient underwent right hemicolectomy and concurrent wedge biopsy followed by intraoperative radiofrequency ablation (RFA) of a single hepatic lesion in segment 7. Thirteen months after surgery, due to multiple new enlarging hepatic metastases, a right hepatectomy was performed with subsequent placement of a hepatic arterial infusion pump (HAIP). Postoperatively, combination floxuridine (FUDR) HAC with six cycles of systemic FOLFOX chemotherapy was administered. Recurrence occurred approximately two years after discontinuation of chemotherapy leading to reinitiation of HAC and administration of irinotecan and cetuximab for a four-month period, which downsized the tumors and enabled a limited hepatic resection of segment 4A. After six months, systemic chemotherapy was resumed to address increasing CEA levels and infiltrative soft tissue at the previous resection margin. For two years, disease control was achieved with different chemotherapy regimens, including cetuximab, panitumumab/irinotecan, and capecitabine. Disease progression led to an additional surgical procedure, consisting of resection of the remainder of segment 4. After surgery the panitumumab/irinotecan regimen was reinitiated but was eventually discontinued due to poor tolerance and poor tumor response.

**Figure 1:**
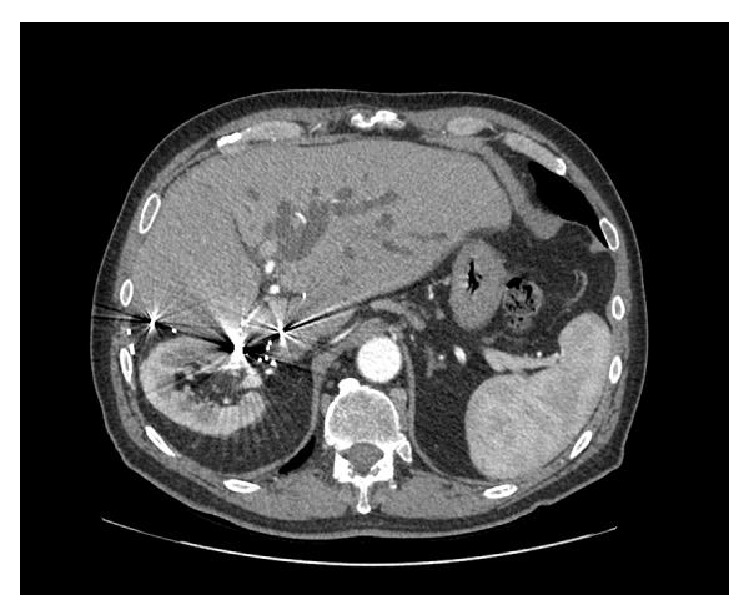
Axial contrast-enhanced CT displaying dilatation of the left hepatic duct due to an enlarging central metastatic lesion.

In the face of progressing hepatic metastases the patient was referred for evaluation regarding appropriateness of SIRT. Bilirubin levels were slightly elevated at presentation (1.6 mg/dL) and for the past six months ranged between 1.3 and 2.3 mg/dL. Imaging revealed several lesions throughout the remnant left liver, including a central lesion causing partial biliary obstruction (Figure 1). Shortly after presentation, bilirubin levels were found to have rapidly increased to 7.5 mg/dL. To address this issue, a 10 × 68 mm Wallstent was primarily placed, under fluoroscopic guidance, across the left hepatic duct obstruction without violation of the papilla. No additional drainage catheters were required and total bilirubin levels following this intervention dropped significantly.

**Figure 2:**
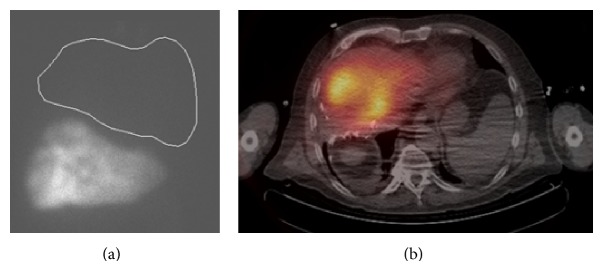
(a) Tc-99 MAA scintigraphy after pre-SIRT mapping demonstrated 6% lung shunting, within acceptable values (<20%). (b) Post-SIRT Bremsstrahlung SPECT/CT showed heterogeneous tracer distribution throughout the remaining left lobe of the liver. No extrahepatic shunting was seen.

With bilirubin levels continuing to drop, the patient was considered a candidate for SIRT and underwent standard evaluation with pre-SIRT arteriography and Tc-99 macroaggregate albumin (MAA) mapping. Angiographic evaluation revealed tumor arterial supply by the left hepatic artery and the right phrenic artery. The latter was embolized with microcoils to redistribute arterial flow to the tumors via the hepatic artery and thus optimize ^90^Y microsphere distribution. Tc-99 MAA hepatic scintigraphy demonstrated 6% lung shunting (Figure 2(a)), within acceptable values (<20%). One month after biliary stent placement and two weeks after the mapping session, serum bilirubin levels were marginally above the upper limit of normal (1.4 mg/dL). SIR-spheres (^90^Y-resin microspheres; Sirtex Medical, Sydney, Australia) were injected selectively in the left hepatic artery through a microcatheter, delivering the entire dose of 52.6 mCi (1.95 GBq). SPECT/CT Bremsstrahlung liver imaging confirmed successful delivery of the full dose within the liver (Figure 2(b)), without any extrahepatic activity. The patient tolerated the procedure well, without any significant acute or delayed side effects/toxicities. Total bilirubin levels during this period did not exceed the levels before SIRT.

**Figure 3:**
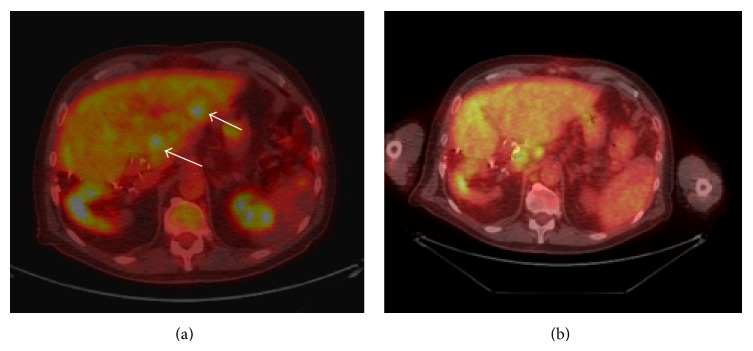
Axial PET-CT images (a) before biliary drainage and SIRT and (b) two months after SIRT, at the level of segments 2 and 3. Significant response to treatment is evident, with resolution of FDG avid hepatic metastases (arrows). However, progression of extrahepatic disease was noted in portocaval and periaortic nodes.

Imaging revealed complete PET response to SIRT and resolution of SUV activity within the liver (Figure 3). Two months after the procedure, despite control of hepatic disease, progression in nodal sites outside the liver and enlarging pulmonary nodules mandated reinitiation of systemic chemotherapy with 5-FU/leucovorin and panitumumab, which temporarily controlled metastatic disease. The patient eventually succumbed to his illness at an outside hospital seven months after SIRT and 10.5 years after initial diagnosis. There was no evident progression of CLM on the latest available CT scan obtained 5 months after SIRT, despite extrahepatic nodal disease involvement.

## 3. Discussion

For patients with unresectable chemorefractory CLM, SIRT has established safety, is well tolerated, and provides promising survival and response rates [1, 6, 7]. Patient selection characteristics for SIRT include, among others, liver-dominant tumor burden, patients with a life expectancy greater than 3 months, adequate hepatic function (serum bilirubin levels <2 mg/dL), and a satisfactory performance status [8]. In this case, the patient had a performance status within the inclusion criteria (ECOG status 0) despite being heavily pretreated for over 10 years with systemic chemotherapy and HAC, as well as with multiple hepatic resections. It has been shown that, in the salvage setting after different chemotherapy regimens or after systemic and HAIP chemotherapy, SIRT is safe provided that bilirubin levels remain below 1.5 mg/dL [2–5]. Biliary intervention (drainage, stenting, or bilioenteric anastomosis) prior to SIRT has been reported and in several institutions is not considered an exclusion criterion [9, 10]. However to our knowledge this is the first reported case of primary biliary stenting as a bridge to SIRT in a patient with coexisting limited hepatic reserve due to multiple resections and HAC. In our patient, it was contemplated that the rapid elevation in bilirubin was primarily due to a centrally located metastasis causing biliary obstruction rather than a consequence of HAC toxicity or of rapid deterioration of hepatic function. The latter is a significant factor to take into consideration when treating patients with hepatocellular carcinoma and underlying cirrhosis, as well as those with metastatic disease and chemotherapy induced steatohepatitis [11].

Whenever feasible, treatment of high level biliary obstruction with primary stent placement avoiding violation of the papilla is recommended. This provides optimal drainage and normalization of bilirubin levels and at the same time minimizes the contamination of the biliary tree by enteric contents and the risk of hepatic abscess formation, as the protective role of the papilla is maintained [12]. The presence of an incompetent sphincter of Oddi is a well-recognized risk factor for hepatic abscess formation after chemoembolization [13], but recent evidence suggests that this risk may be lower for SIRT [9]. To prevent the development of cholangitis and abscess formation in this patient, two intravenous doses of cefotetan were administered before and after biliary stenting. Prior to SIRT, an additional dose of cefotetan was administered and in the postprocedural setting the patient received prophylaxis with oral metronidazole and ciprofloxacin for five days.

Despite the fact that the patient had limited hepatic reserve due to previous hepatectomies, the entire liver remnant was treated with administration of the full calculated ^90^Y dose within the left hepatic artery without stasis. This approach may be followed when treating patients with limited hepatic volume, although a conservative approach with sequential lobar treatment is preferred when treating patients with bilobar disease [2, 14]. After the procedure and during follow-up evaluation there was no evidence of significant toxicity, liver failure, or radioembolization induced liver disease (REILD), even though the patient had risk factors for the development of these complications (treatment of the entire liver remnant, elevated baseline bilirubin levels, and multiple chemotherapy regimens) [15, 16].

Although at least in theory patients who receive HAC should not require any arterial embolization for flow redistribution, it is noteworthy that extrahepatic collaterals or accessory gastric or pancreaticoduodenal branches can recanalize and supply the hepatic tumors. This was the case in this patient with phrenic arterial recruitment supplying tumor. Rarely after HAIP placement or coil embolization for prior SIRT sessions, collaterals can still develop or previously ligated/embolized vessels may recanalize, requiring additional embolization for safe and optimal delivery of the ^90^Y microspheres [17].

In conclusion, the current case demonstrates that, with appropriate evaluation and biliary intervention, SIRT can still be safe and effective in the most compromised patients, even in the face of biliary obstruction.
